# Supplementary material for: Atomic-layer Rashba-type superconductor protected by dynamic spin-momentum locking
Source: Nat Commun. 2021 Mar 5;12:1462. doi: 10.1038/s41467-021-21642-1 (PMC7935850; doi:10.1038/s41467-021-21642-1)
Supplement: Supplementary file 1 — Supplementary Information [file 41467_2021_21642_MOESM1_ESM.pdf]

Supplementary Information for

Atomic-layer Rashba-type superconductor protected by dynamic spin-momentum locking

Shunsuke Yoshizawa, Takahiro Kobayashi, Yoshitaka Nakata, Koichiro Yaji, Kenta Yokota, Fumio Komori, Shik Shin, Kazuyuki Sakamoto, and Takashi Uchihashi

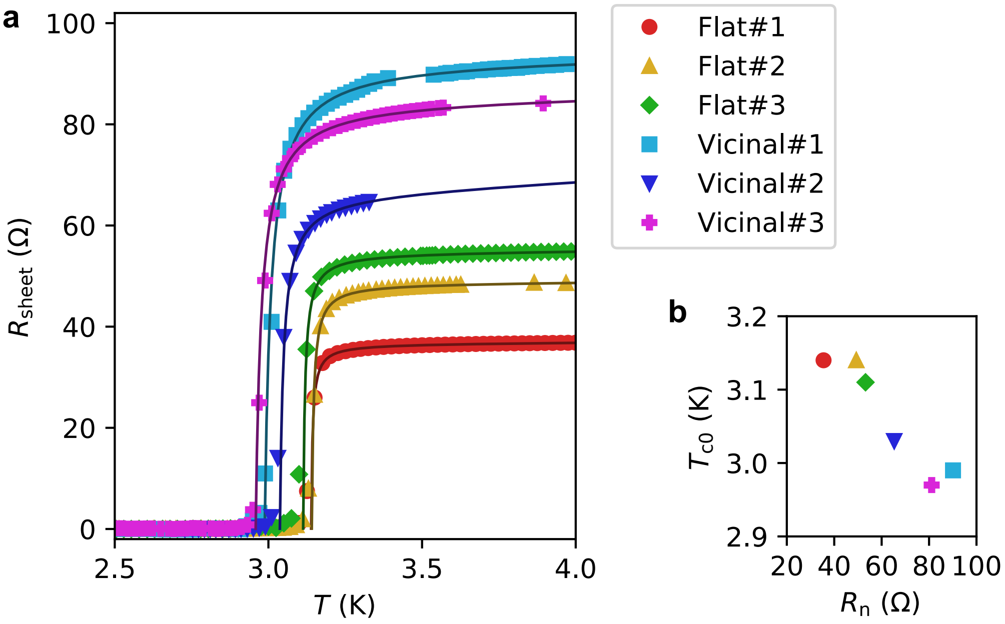

Supplementary Figure 1 | Determination of zero-field critical temperature. (**a**) Resistance curves of the six samples (Flat#1, Flat#2, Flat#3, Vicinal#1, Vicinal#2, and Vicinal#3) measured in zero magnetic fields are plotted. The solid curves show the results of the fitting. (**b**) Zero-field critical temperature (T_{c0}) plotted as a function of normal-state registance (R_n).

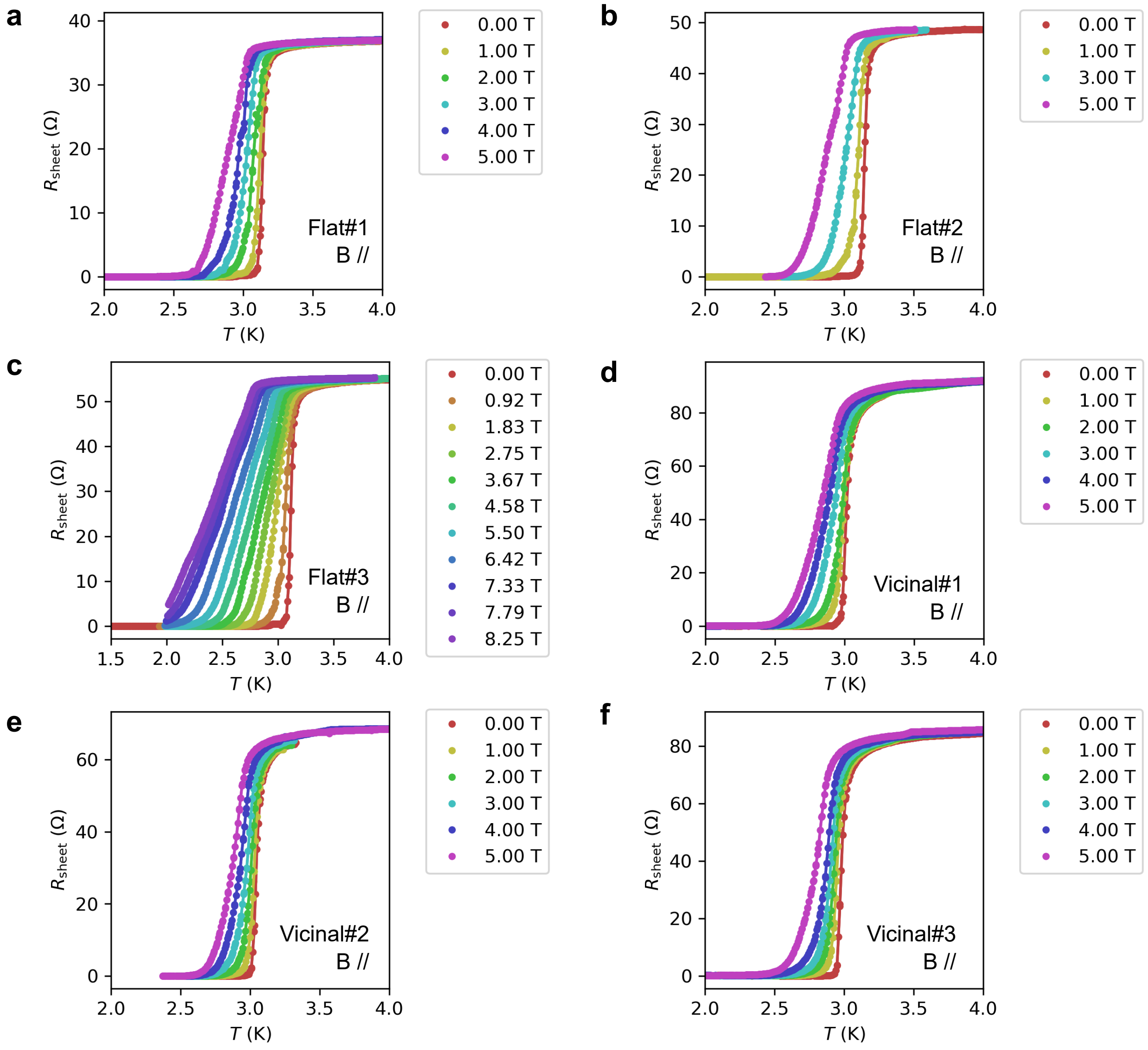

Supplementary Figure 2 | Superconducting transition in in-plane magnetic fields. (**a-f**) Resistance curves of the six samples (Flat#1, Flat#2, Flat#3, Vicinal#1, Vicinal#2, and Vicinal#3) in in-plane magnetic fields.

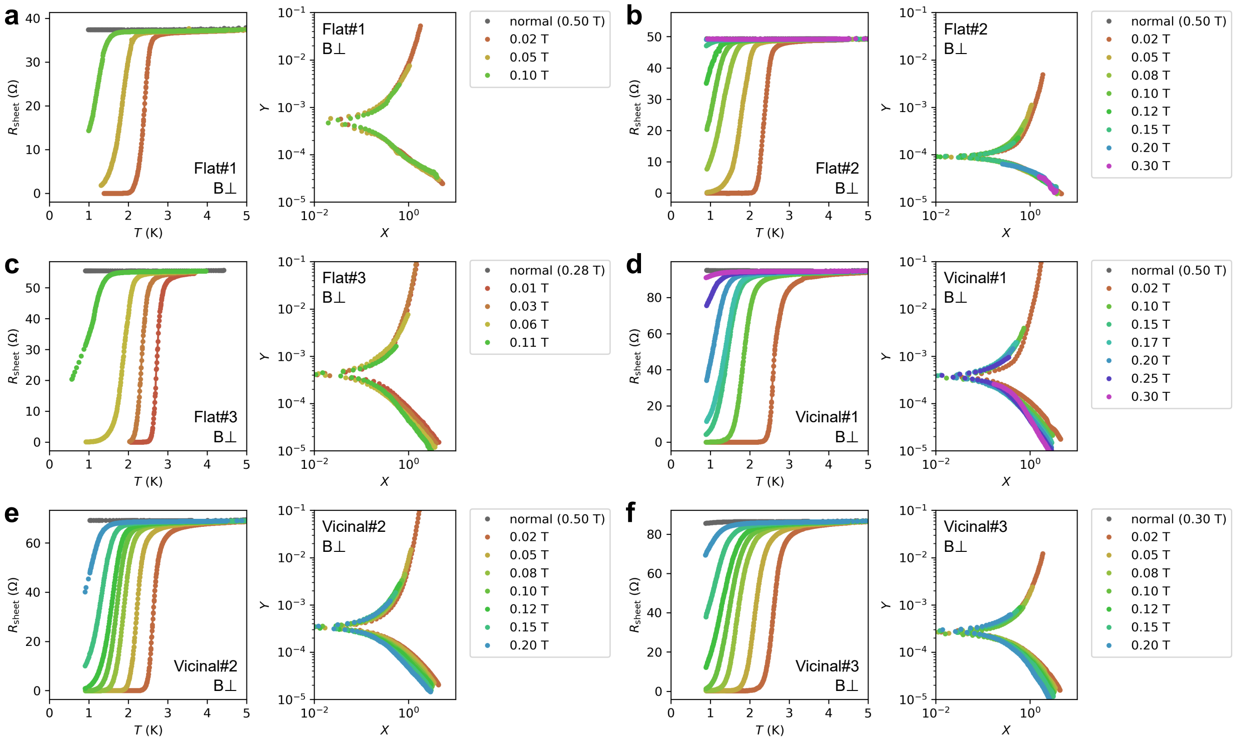

Supplementary Figure 3 | Superconducting transition in out-of-plane magnetic fields. (**a**-**f**) Resistance curves (left) and the plots of the Ullah-Dorsey scaling analysis[1,2] (right) of the six samples (Flat#1, Flat#2, Flat#3, Vicinal#1, Vicinal#2, and Vicinal#3) in out-of-plane magnetic fields. The axes of the scaling plot are $X = |T - T_c|(BT)^{-1/2}$ and $Y = (B/T)^{1/2}$.

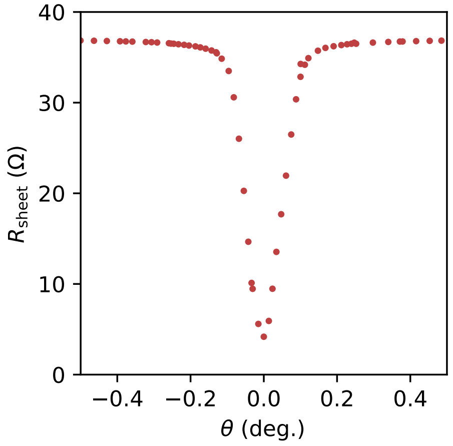

Supplementary Figure 4 | Accuracy in the sample alignment for in-plane magnetic fields. The plot shows the typical angular dependence of the sheet resistance near the critical temperature. Here, R_{sheet} was measured at $T = 2.8$ K in $B = 5$ T. The sample angle θ was determined from the signal of the Hall sensor attached on the rotatable sample stage. The parallel sample configuration was found from the minimum of the curve by changing θ at an accuracy of ~0.05° for each sample.

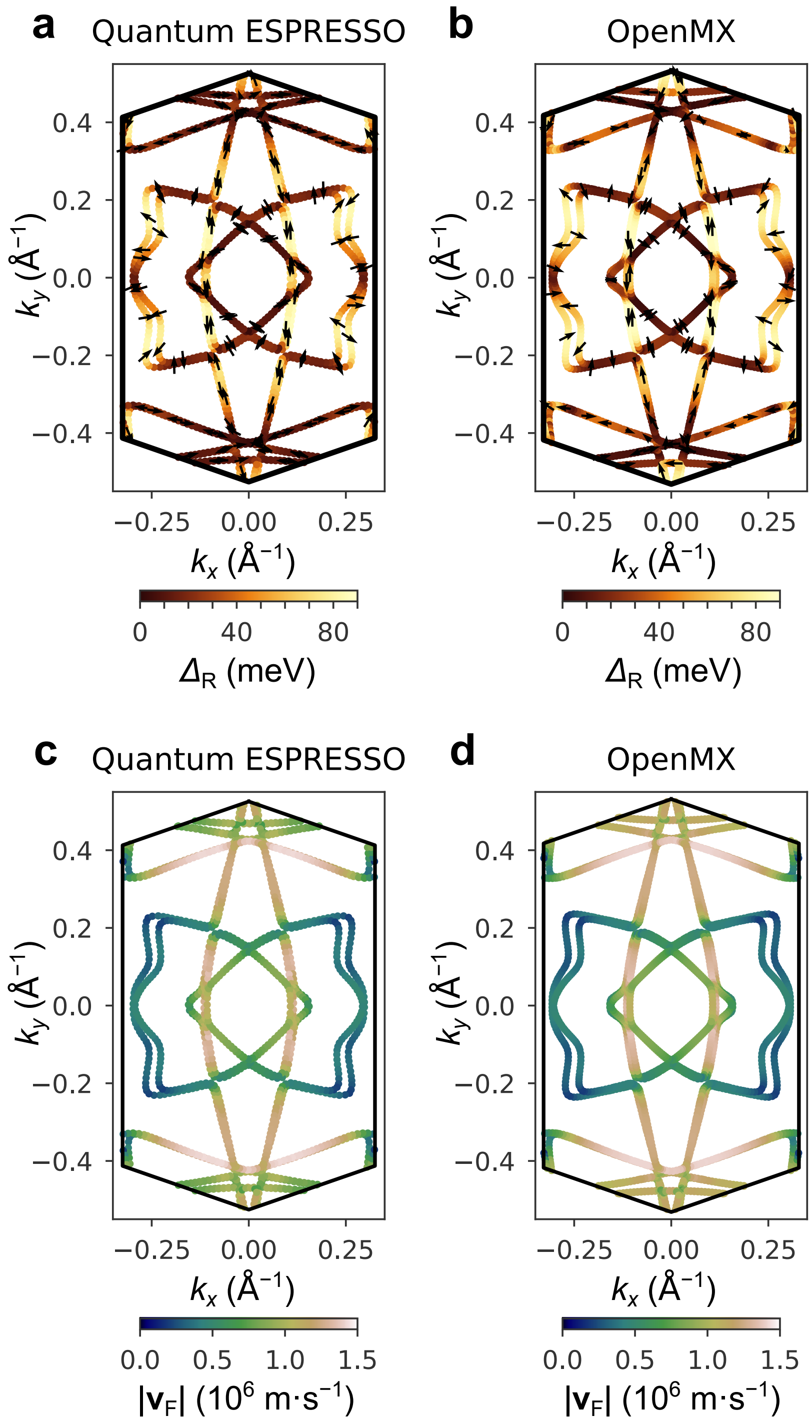

Supplementary Figure 5 | Comparison of calculations using Quantum ESPRESSO and OpenMX. (**a**,**b**) Band splitting and spin polarisation direction of $\sqrt{7} \times \sqrt{3}$-In calculated with (**a**) Quantum ESPRESSO and (**b**) OpenMX. (**c**,**d**) Fermi velocity calculated with (**c**) Quantum ESPRESSO and (**d**) OpenMX.

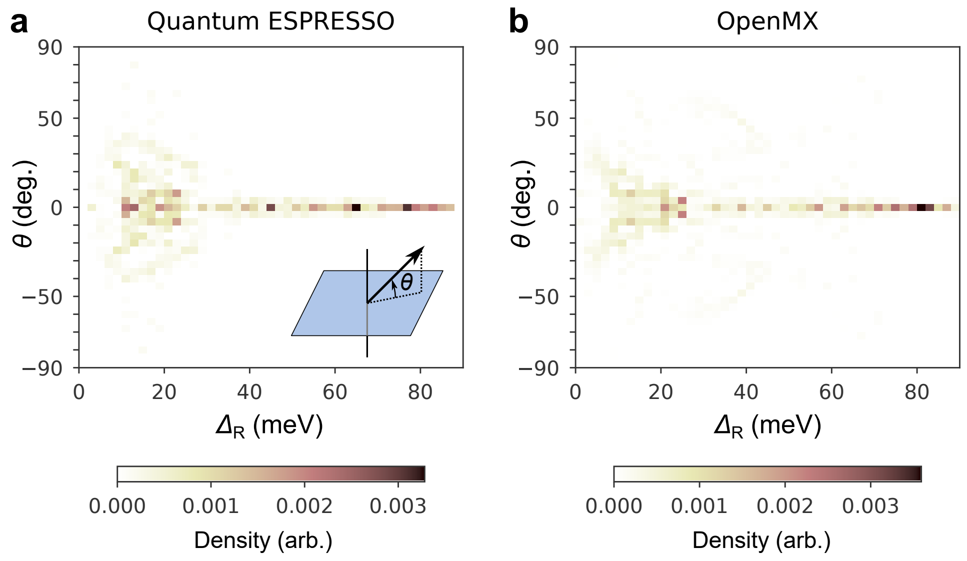

Supplementary Figure 6 | Spin polarizaton direction on the Fermi surface. Spin orientation relative to the in-plane direction is displayed as a function of energy splitting. The results are obtained using (**a**) Quantum ESPRESSO and (**b**) OpenMX. $\theta = 0°$ corresponds to the in-plane direction while $\theta = 90°$ the out-of-plane direction (see the inset of **a**).

Supplementary Note 1 | Fitting analysis of zero-field data

The zero-field critical temperature T_{c0} and the normal-state residual resistance R_n were determined by fitting a function of the form[3],

$$\frac{1}{R_{\mathrm{sheet}}} = \frac{1}{R_n + cT^a} + \frac{1}{R_0}\frac{T}{T - T_{c0}} \tag{1}$$

to the resistance curves (Supplementary Figure 1a) for $T > T_{c0}$. Here, the first term represents normal-state conductance; a and c represent phonon contribution; the second term represents the fluctuation effect characteristic to 2D superconductors[4]; and R_0 is the temperature-independent parameter. As presented in Supplementary Figure 1b, T_{c0} is found to be a monotonic decreasing function of R_n, being order-of-magnitude consistent with the theoretical behaviour of superconducting films containing non-magnetic impurities[5]. This consistency suggests that the atomic steps work as non-magnetic scatterers in the present condition, while it serves as Josephson junctions when the supercurrent density approaches the critical value[6,7]. The weak dependence of T_{c0} on R_n indicates that the s-wave pairing is dominant.

Supplementary Note 2 | Analysis of pair-breaking mechanism in out-of-plane fields

The linear B dependence of T_c observed for out-of-plane fields (Fig. 5d, which was obtained from the scaling analysis shown in Supplementary Figure 3) is consistent with the Ginzburg-Landau (GL) theory[8]. We estimate the extrapolated value of GL coherence length, ξ, by fitting the following function valid near T_{c0},

$$B = \frac{\Phi_0}{2\pi\xi^2}\left(1 - \frac{T_c}{T_{c0}}\right) \tag{2}$$

where $\Phi_0 = h/(2e)$ is the flux quantum. As presented in Table 1, ξ ranges between 29-43 nm, which are consistent with the radii of vortices (36-47 nm) reported in STM studies[7]. This indicates that the perpendicular critical field is limited by the orbital pair-breaking effect, i.e., penetration of vortices.

Supplementary Note 3 | The influence of the static effect of the Rashba-type SOC

The static effect of the spin-momentum locking due to the Rashba-type SOC is known to enhance the in-plane critical field $B_{c2\parallel}$ by a factor of 2 from the Pauli limit. This effect is likely to be

weakened by electron scattering and mixing between different spin states. Since we do not know the degree of such an effect, we assume the worst case and estimate the upper limit of error in τ_s in the following.

In the absence of spin-orbit coupling, the B dependence of T_c near T_{c0} is given by

$$1 - \frac{T_c}{T_{c0}} = \frac{7\zeta(3)}{4\pi^2} \cdot \frac{(\mu_B B)^2}{(k_B T_{c0})^2}. \tag{3}$$

With the static spin-momentum locking due to Rashba SOC, the expression changes to[9]

$$1 - \frac{T_c}{T_{c0}} = \frac{1}{2} \cdot \frac{7\zeta(3)}{4\pi^2} \cdot \frac{(\mu_B B)^2}{(k_B T_{c0})^2}. \tag{4}$$

Here the addition of the factor 1/2 in Eq. (4) means that B is replaced with an effective magnetic field $B_{\text{eff}} = (1/\sqrt{2})B$ in Eq. (3). This is the origin of the enhancement of B_{c2} by a factor of $\sqrt{2}$ due to the static locking effect of Rashba SOC. The effect of non-magnetic disorder on a Rashba superconductor can be estimated using this effective magnetic field. By substituting B with $B_{\text{eff}} = (1/\sqrt{2})B$ in the following equations (taken from Eqs. (3) and (6) in the main text; only the paramagnetic contribution is considered here),

$$\alpha(B) = c_P B^2 \tag{5}$$

and

$$c_P = \frac{3\tau_s \mu_B^2}{2\hbar}. \tag{6}$$

We see that τ_s value is doubled for the same experimental data $\alpha(B)$. With the τ_s values obtained previously (see Table 1) in the manuscript, we can estimate that the lower limit of τ_{el}/τ_s is 0.25-0.5. These values are still much higher than 1/60-1/1000 for thin In films, which is due to the atomistic spin-orbit scattering mechanism. Therefore, the result is not attributable only to the conventional mechanism, and our conclusion remains the same.

Supplementary Note 4 | The role of the Zeeman-type SOC in the B_{c2} enhancement

The distribution of spin polarisation direction was obtained from our DFT results (Supplementaray Figure 6a). It clearly shows that the spins align in the in-plane directions for the most of energy regions. This means that Rashba SOC is dominant over Zeeman SOC mostly. The spins tend to tilt toward the out-of-plane direction below 30 meV, but the off-angle is about 45° at most.

Namely, there is no region where Zeeman SOC is dominant. This result was also reproduced by the calculations using OpenMX (Supplementaray Figure 6b).

This non-dominant Zeeman SOC confined to small area of the Fermi surface can barely enhance B_{c2}. This is because enhancement factor is determined by an average over the whole Fermi surface. The critical temperature in the presence of magnetic field B, T_c(B), is given by the following equations[10,11],

$$\ln\left(\frac{T_c(\mathbf{B})}{T_{c0}}\right) = 2\left\langle |\psi(\mathbf{k})|^2 f\left(\frac{\boldsymbol{g}(\mathbf{k})\cdot\mathbf{B}}{\pi T_c|\boldsymbol{g}(\mathbf{k})|}\right)\right\rangle_{\mathbf{k}} \tag{7}$$

and

$$f(x) = \mathrm{Re}\sum_{n=1}^{\infty}\left(\frac{1}{2n-1+ix} - \frac{1}{2n-1}\right), \tag{8}$$

where $T_{c0} \equiv T_c(\mathbf{B} = 0)$, $\mathbf{k}$ is the wavevector on the Fermi surface, $\psi(\mathbf{k})$ is the spin-singlet gap function, $\boldsymbol{g}(\mathbf{k})$ is a vector determining the spin polarisation at each $\mathbf{k}$, and $\langle \ldots \rangle_{\mathbf{k}}$ denotes taking an average over the Fermi surface. B_{c2} is given by $\mathbf{B}$ that satisfies $T_c(\mathbf{B}) = 0$. For example, when magnetic field is applied parallel to the Rashba-split Fermi surface, spins in some regions point nearly perpendicular to the field (see the figure (a) below). This is analogous to the Zeeman SOC case (see the figure (b) below), but the enhancement factor is limited to 2 because of the averaging over the whole Fermi surface. This clearly shows that, even if Zeeman SOC coexists in the present system and tilts some of the spins toward the out-of-plane direction, its effect is limited.

If the dynamics of spins is considered, the effect of the Zeeman-type SOC can be suppressed even more. Since our system is characterised by dominant Rashba-type SOC, the polarisation axis of the spins strongly depends on momentum. This means that electron scattering between different momenta effectively induce a spin flipping and therefore out-of-plane spin component is not conserved through elastic scattering. We note that a different situation takes place for an ultrathin layer of TMDC with Zeeman-type SOC[12,13]. Here, since all the spins around one of the valleys are polarised in the same out-of-plane direction, dominant intravalley scatterings cannot cause spin flipping[14]. By contrast, this mechanism does not apply to our Rashba-type SOC case because of the presence of frequent spin flipping.

Base on all these facts, we conclude that strong enhancement of B_{c2} due to Zeeman SOC is extremely unlikely in the present system.

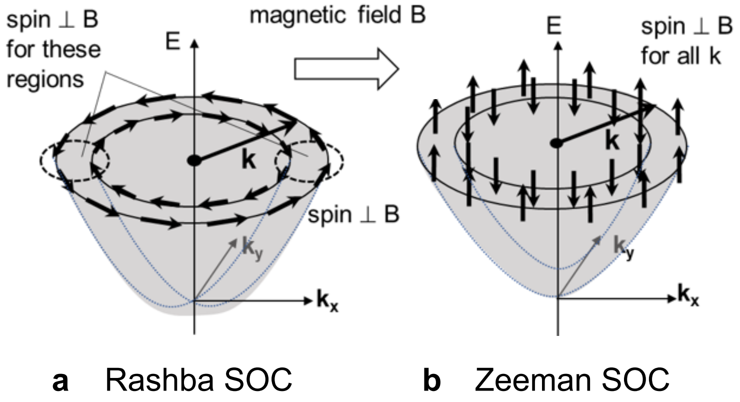

Schematic illustration of the Fermi surface splitting due to (a) Rashba SOC and (b) Zeeman SOC.

Supplementary Note 5 | Computational condition for OpenMX

We performed DFT calculations using OpenMX to confirm the reproducibility of the computational results. OpenMX is based on the optimised pseudo-atomic orbitals (PAO)[15], and we selected from the "Precise" sets from the PAO database (2019) in the OpenMX website[16]. We chose LSDA-CA[17,18] for the exchange-correlation functional. The crystal structure of $\sqrt{7} \times \sqrt{3}$-In was modeled by a repeated slab consisting of an In bilayer, nine Si bilayers, a H layer for termination, and a vacuum region of 4.5 nm. We set the cutoff energy to 300 Ry and the k-point mesh to $6 \times 8 \times 1$. The geometry optimisation was performed without SOC until the maximum force on each atom became less than 1×10^{-5} Hartree·Bohr^{-1}. The band calculation was carried out using the optimised structure by including SOC. The spin texture was analysed by using kSpin code[19].

Supplementary References

1. Ullah, S. & Dorsey, A. T. Effect of fluctuations on the transport properties of type-II superconductors in a magnetic field. *Phys. Rev. B* **44**, 262–273 (1991).
2. Saito, Y., Nojima, T. & Iwasa, Y. Quantum phase transitions in highly crystalline two-dimensional superconductors. *Nat. Commun.* **9**, 778 (2018).
3. Uchihashi, T., Mishra, P. & Nakayama, T. Resistive phase transition of the superconducting Si(111)-($\sqrt{7} \times \sqrt{3}$)-In surface. *Nanoscale Res. Lett.* **8**, 167 (2013).
4. Aslamasov, L. G. & Larkin, A. I. The influence of fluctuation pairing of electrons on the conductivity of normal metal. *Phys. Lett. A* **26**, 238–239 (1968).

5. Finkel'stein, A. M. Suppression of superconductivity in homogeneously disordered systems. *Physica B: Condensed Matter* **197**, 636–648 (1994).

6. Uchihashi, T., Mishra, P., Aono, M. & Nakayama, T. Macroscopic superconducting current through a silicon surface reconstruction with indium adatoms: Si(111)-($\sqrt{7} \times \sqrt{3}$)-In. *Phys. Rev. Lett.* **107**, 207001 (2011).

7. Yoshizawa, S. *et al.* Imaging Josephson vortices on the surface superconductor Si(111)-($\sqrt{7} \times \sqrt{3}$)-In using a scanning tunneling microscope. *Phys. Rev. Lett.* **113**, 247004 (2014).

8. Tinkham, M. *Introduction to superconductivity* (Dover, 2004).

9. Barzykin, V. & Gor'kov, L. P. Inhomogeneous stripe phase revisited for surface superconductivity. *Phys. Rev. Lett.* **89**, 227002 (2002).

10. Frigeri, P. A., Agterberg, D. F., Koga, A. & Sigrist, M. Superconductivity without inversion symmetry: MnSi versus $CePt_3Si$. *Phys. Rev. Lett.* **92**, 097001 (2004).

11. Smidman, M., Salamon, M. B., Yuan, H. Q. & Agterberg, D. F. Superconductivity and spin–orbit coupling in non-centrosymmetric materials: a review. *Rep. Prog. Phys.* **80**, 036501 (2017).

12. Lu, J. M. *et al.* Evidence for two-dimensional Ising superconductivity in gated MoS_2. *Science* **350**, 1353–1357 (2015).

13. Saito, Y. *et al.* Superconductivity protected by spin-valley locking in ion-gated MoS_2. *Nat. Phys.* **12**, 144–149 (2016).

14. Ilić, S., Meyer, J. S. & Houzet, M. Enhancement of the upper critical field in disordered transition metal dichalcogenide monolayers. *Phys. Rev. Lett.* **119**, 117001 (2017).

15. Ozaki, T. Variationally optimized atomic orbitals for large-scale electronic structures. *Phys. Rev. B* **67**, 155108 (2003).

16. Ozaki, T. *et al.* OpenMX: open source package for Material eXplorer. `http://www.openmx-square.org/` (2019).

17. Ceperley, D. M. & Alder, B. J. Ground state of the electron gas by a stochastic method. *Phys. Rev. Lett.* **45**, 566–569 (1980).

18. Perdew, J. P. & Zunger, A. Self-interaction correction to density-functional approximations for many-electron systems. *Phys. Rev. B* **23**, 5048–5079 (1981).

19. Kotaka, H., Ishii, F. & Saito, M. Rashba effect on the structure of the Bi one-bilayer film: fully relativistic first-principles calculation. *Jpn. J. Appl. Phys.* **52**, 035204 (2013).
